# Influence of long-term equine herpesvirus type 1 (EHV-1) infection on primary murine neurons—the possible effects of the multiple passages of EHV-1 on its neurovirulence

**DOI:** 10.1007/s12223-017-0528-5

**Published:** 2017-04-13

**Authors:** Joanna Cymerys, A. Słońska, A. Tucholska, A. Golke, A. Chmielewska, M. W. Bańbura

**Affiliations:** Division of Microbiology, Department of Preclinical Sciences, Faculty of Veterinary Medicine, Warsaw University of Life Sciences – SGGW, Ciszewskiego 8, 02-786 Warsaw, Poland

## Abstract

Equine herpesvirus 1 (EHV-1), like other members of the *Alphaherpesvirinae* subfamily, is a neurotropic virus causing latent infections in the nervous system of the natural host. In the present study, we have investigated EHV-1 replication (wild-type Jan-E strain and Rac-H laboratory strain) during long-term infection and during the passages of the virus in cultured neurons. The studies were performed on primary murine neurons, which are an excellent in vitro model for studying neurotropism and neurovirulence of EHV-1. Using real-time cell growth analysis, we have demonstrated for the first time that primary murine neurons are able to survive long-term EHV-1 infection. Positive results of real-time PCR test indicated a high level of virus DNA in cultured neurons, and during long-term infection, these neurons were still able to transmit the virus to the other cells. We also compared the neurovirulence of Rac-H and Jan-E EHV-1 strains after multiple passages of these strains in neuron cell culture. The results showed that multiple passages of EHV-1 in neurons lead to the inhibition of viral replication as early as in the third passage. Interestingly, the inhibition of the EHV-1 replication occurred exclusively in neurons, because the equine dermal (ED) cells co-cultivated with neuroculture medium from the third passage showed the presence of large amount of viral DNA. In conclusion, our results showed that certain balance between EHV-1 and neurons has been established during in vitro infection allowing neurons to survive long-term infection.

## Introduction

The natural biology of alphaherpesviruses is a primary infection with mild or no symptoms and a highly successful establishment of a long-term relationship with the host. Alphaherpesviruses (e.g. HSV-1, HSV-2, BHV-1, VZV, PRV) establish a life-long latency in peripheral neurons where productive replication is suppressed. Equine herpesvirus 1 (EHV-1), a major causative agent of upper respiratory tract infections and abortion in horses, similarly to other alphaherpesviruses is also neurotropic and causes latent infection in the neurons of natural host (Delhon et al. 2002; Ch’ng and Enquist 2005). Despite the fact that many studies have been devoted to the pathogenesis of various clinical forms of EHV-1 infection, mechanisms of the neuronal damage are not fully understood (Pusterla and Hussey 2014; Slater et al. 1994; Sauerbrei and Wutzler 2002; De Regge et al. 2006; Bańbura et al. 2000). Most of the available information about the latency establishment, maintenance and reactivation of alphaherpesviruses is derived from in vivo studies (Wilson and Mohr 2012; Sauerbrei and Wutzler 2002; Baxi 1995); however, it is difficult to differentiate specific effects of direct virus-neuron relationship from indirect consequences mediated by immune or non-neuronal supportive cells. Consequently, presented in vitro model utilizing cultured primary murine neurons provides a simple and effective method to examine the kinetics of EHV-1 replication, to determine the differences in the influence of the virus on the neuronal cell depending on virus strain and its adaptation to the cell culture. Our previous studies had provided answers to a number of key EHV-1-related questions. We found out that the field isolate Jan-E (at low in vitro passage) and reference strain Rac-H (at high in vitro passage) were able to replicate without the need for adaptation in murine neurons in vitro. Moreover, positive real-time PCR (quantitative PCR; qPCR) and *nested*PCR (nPCR) results indicated the presence of viral DNA in neurons 24–48 h post infection (p.i.) with both EHV-1 strains; however, some neurons survived infection and showed EHV-1 replication (Cymerys et al. 2010). We also demonstrated that Rac-H and Jan-E EHV-1 strains were able to induce apoptosis in primary murine neurons; however, apoptotic neurons represented only a small percentage of the total cell number. Moreover, the majority of the observed neurons were not only resistant to the EHV-1-induced cell death but also re-entered the active proliferation cycle (increase in the number of cells in S and G2M phases of cell cycle) (Cymerys et al. 2012). We also observed an autophagy induction during infection of primary murine neurons with both Jan-E and Rac-H strains of EHV-1. However, chloroquine (autophagy inhibitor) did not affect the level of replication of the virus, quantified with real-time PCR method (Cymerys et al. 2014). These results led us to further studies on kinetics of EHV-1 replication during long-term neuronal infection and to examine the effects of the neuronal passage on EHV-1 neurovirulence.

## Materials and methods

### Virus strains

In the present study, two strains of EHV-1 from the virus collection of the Virology Laboratory of the Department of Preclinical Sciences were used. Jan-E is a field strain isolated from aborted foetus (mare Ezelda, Poland; 12th passage in ED cells) and identified by PCR using gB-specific primers (Borchers and Slater 1993). Rac-H is a reference strain isolated in 1957 from the aborted foetus of mare Heraldia, Poland. This strain has been passaged through a series of cell cultures and nowadays is defined as ‘pantropic, non-pathogenic’ (Nugent et al. 2006).

### Neuronal cell cultures

Balb/c (H-2^d^) mice genetically susceptible to EHV-1 infection (Awan et al. 1990; Gosztonyi et al. 2009) were used to establish primary culture of murine neurons, as described before (Cymerys et al. 2010). Cells were plated onto poly-l-lysine or poly-d-lysine with laminin-coated coverslides at a density of 5 × 10 (Baxi et al., 1995) to 10^4^ neurons per well (9.6 cm^2^) and into cell culture flasks (10^4^ cells per 25cm^2^ flask). Primary murine neurons were cultured in B-27 Neuron Plating Medium (Gibco Life Technologies), consisting of neurobasal medium, B27 supplement, glutamine (200 mmol/L), glutamate (10 mmol/L; 1.8 mg/mL), antibiotics (penicillin-streptomycin, 1%) with 10% supplement of foetal bovine (5%) and equine serum (5%) (Gibco Life Technologies), and maintained at 37 °C with 5% CO_2_. Four days after plating, the medium was removed and replaced with Neuron Feeding Medium (B-27 Neuron Plating Medium without glutamate). In this medium, neurons were maintained for the next 6 days prior to further manipulations.

### Inoculation of cells

Primary cultures of murine neurons (10^5^ cells per 9.6 cm^2^ well) were infected with EHV-1 (MOI = 1.0) for 60 min at 37 °C. After adsorption, the inoculum were removed and replaced with fresh culture medium. Infected cells were incubated for 24, 48, 72, 96, 120, 144 and 168 h and 14 or 21 days at 37 °C with 5% CO_2_.

### Real-time cell growth analysis

Cellular growth, behaviour and morphology of neuronal cells infected with EHV-1 strains were analyzed by JuLI™ Br Live Cell Analyzer—system for bright-field analysis (NanoEnTek, Korea 2015). Neuronal cells (10^5^ cells per 9.6 cm^2^ well) were seeded in a 6-well plate and infected with Jan-E or Rac-H EHV-1 strains as described above. Cell-growth images were captured for 160 h with 7-min interval. Cell confluence analysis, as well as real-time cell growth curve, was generated using JuLI Br PC software. Uninfected cells were used as a negative control. All images were captured at a ×40 magnification.

### Passage of virus in primary culture of murine neurons

Primary cultures of murine neurons (10^5^ cells per 25cm^2^ flask) were infected with EHV-1 (MOI = 1.0) (Rac-H or Jan-E strain) for 60 min at 37 °C. After adsorption, the inoculum was removed and replaced with fresh culture medium. Subsequently, cells were incubated for 48 h at 37 °C with 5% CO_2_ and then infected neurons were frozen in −20 °C (passage 0). The cultures were then thawed and the lysates used for infection of fresh 7-day-old cultures of murine neurons (passage I). The entire cell lysate from passage 0 was used as inoculum. Following 60 min of adsorption at 37 °C, the inoculum was removed, replaced with fresh culture medium, and the cultures were maintained at 37 °C in a humidified atmosphere with 5% CO_2_ for 48 h. This process was repeated for further nine passages. Neuronal cultures infected directly with the EHV-1 (Jan-E or Rac-H strain), propagated in ED cell cultures (equine dermal cell line; American Tissue Culture Collection, ATCC No. CCL57), served as positive controls. Uninfected neurons served as negative controls.

### Real-time PCR

The quantity of the EHV-1 DNA in all samples was estimated using quantitative real-time PCR (qPCR) with fluorescent TaqMan probes, complementary to a sequence within the amplified products. Samples were collected at 1, 2, 6, 7, 11, 14 and 21 days p.i. and during multiple passages of EHV-1 strains in neurons. Viral DNA was isolated separately from cells and cell culture medium, using High Pure Viral Nucleic Acid Kit® (Roche Diagnostics), as instructed by the manufacturer. Real-time PCR tests were run on LightCycler 2.0 instrument (Roche Diagnostics, Germany), using a modified *in-house* quantitative method, described below (Dzieciątkowski et al. 2009). Highly conservative region encoding glycoprotein B (gB) gene has been chosen, and set of primers was developed, as well as the probe, labelled with fluorophore reporter JOE on 5′ end and with BHQ-2 quencher on its 3′ end (Oligo, Poland). Investigations were performed using reaction mixture TaqMan Master Kit^®^ (Roche Diagnostics, Germany). Each amplification reaction embraced, except tested samples, also positive calibrators in range 100–1,000,000 copies per mL and negative control of DNA extraction and amplification process. In order to assess the sensitivity of assay, plasmid construct was developed by cloning the fragment of EHV-1 gB gene (328 bp) into *Sma*I digested pBluescript II SK(−) (Epoch LifeScience, USA). The concentration of obtained amplicon DNA was determined spectrophotometrically by absorbance of UV light at 260 and 280 nm. The limit of detection (LOD) of modified qPCR assay was determined by analysis of serial decimal dilutions of amplicon DNA in range 10^1^–10^6^ copies. Each dilution was prepared and analyzed in six independent replications. Probit analysis was used to calculate the LOD concentration (Burns and Valdiva 2008). Fluorescence levels were measured at 560-nm wavelength, and a threshold cycle (*Ct*) value for each sample was calculated. *Ct* values of EHV-1 calibrators were the basis for standard curves, and the copy numbers were calculated automatically by a software package for data analysis. LOD of used qPCR assay, established on level 227 copies per mL, was adopted for this study as a *cut-off* value.

### Statistical analysis

The results were statistically evaluated by one-way analysis of variation (ANOVA) using the Student-Newman-Keuls multiple comparisons test and the Turkey-Kramer multiple comparisons test by GraphPad PrismTM version 4.03 software (GraphPad Software Inc., San Diego, CA, USA). All experiments were repeated at least three times. Statistical differences were interpreted as significant at *P* ≤ 0.05 (*), highly significant at *P* ≤ 0.01 (**) and not significant at *P* ≥ 0.05.

## Results

### Morphology of neurons infected with EHV-1

**Fig. 1:**
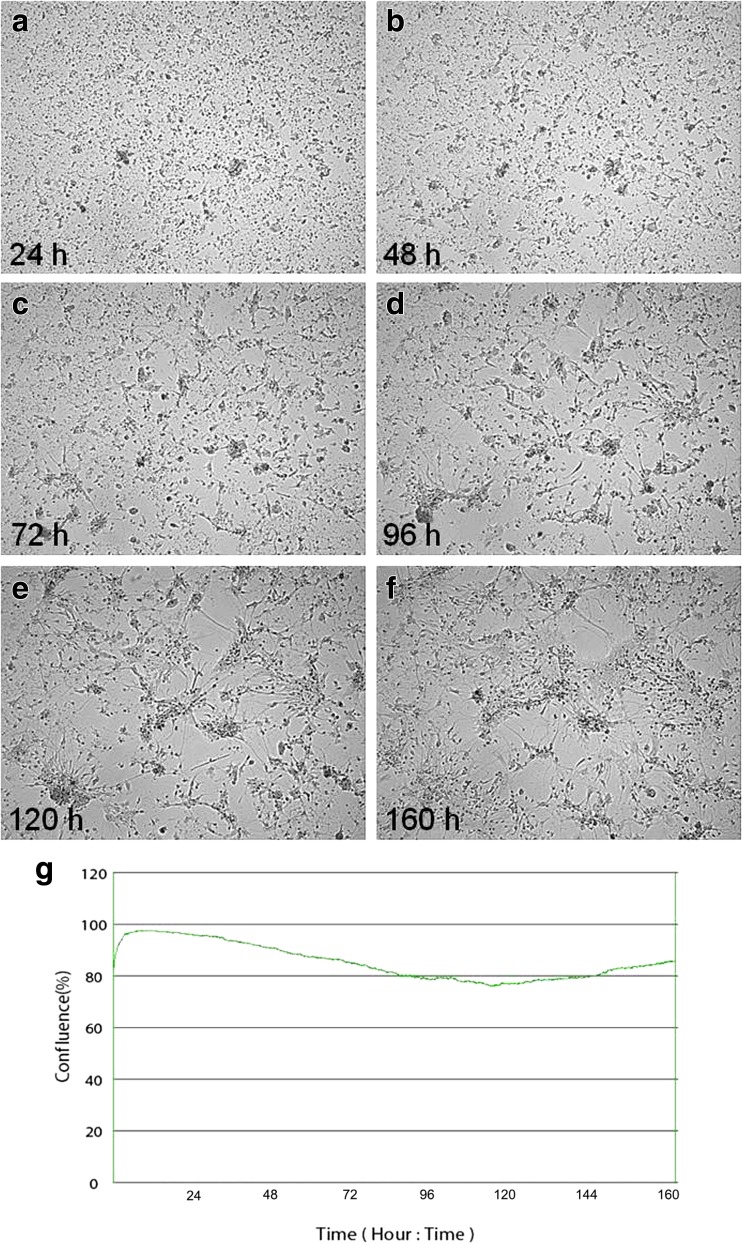
Morphology of uninfected primary murine neurons. Real-time cell growth analysis was performed using live image move analyzer JuLI™ Br. Cultures were observed from initial seeding for 160 h (**a**–**f**). All images were recorded every 7 min and analyzed monolayer confluence (**g**). Objective magnification ×40

**Fig. 2:**
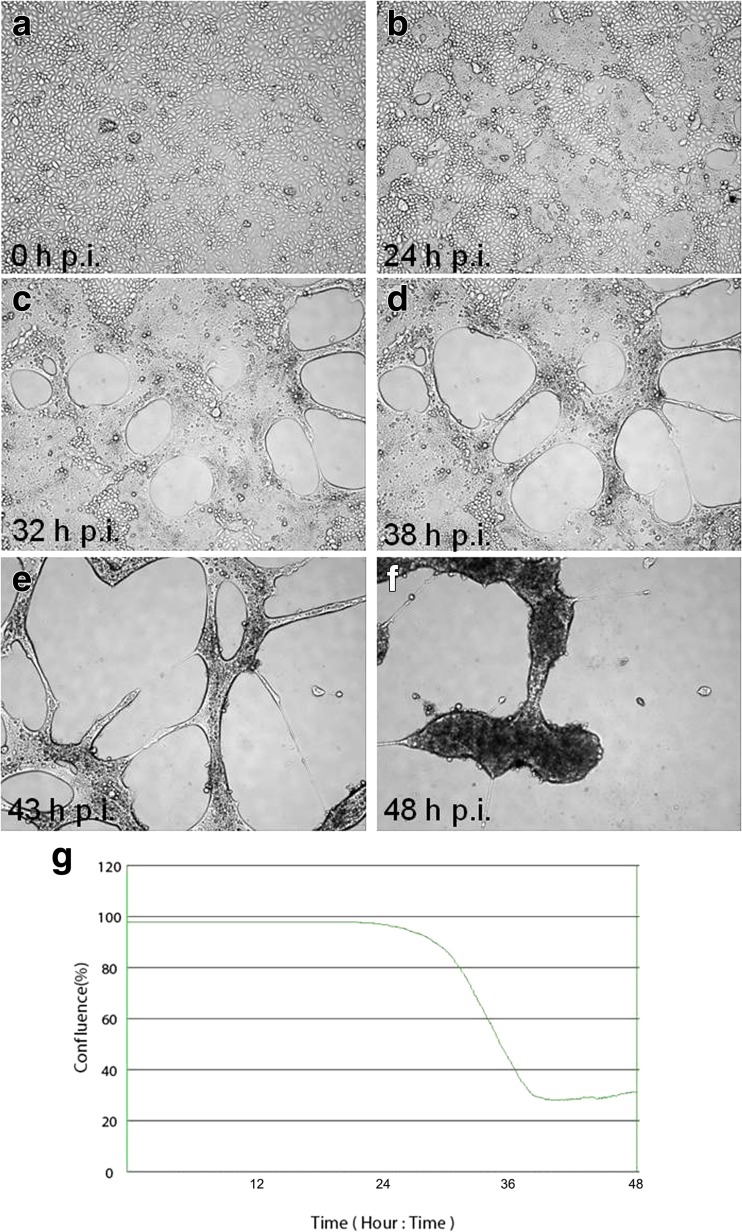
Morphological changes of ED cells infected with Jan-E EHV-1 strain. Cells were observed for 48 h through live image move analyzer JuLI™ Br (**a**–**f**). CPE was manifested by cell destruction and focal degeneration, confirmed by growth curve (**g**). Objective magnification ×40

Real-time cell growth analysis was conducted using live cell movie analyser JuLI Br. During the first 24 h (Fig. 1a), large numbers of adherent neuronal cell bodies were observed. Development of neuronal projections and axon branching were noticed at 72 h (Fig. 1c). During imaging 96–160 h (Fig. 1d–f), neurons continued to extend and formed a dense, intact fibre network, radiating from the neuronal cell bodies through the dendrites and axon. This observation was confirmed by detecting cell confluence and creating growth curve using image base analysis (Fig. 1g). Similar analyses were conducted for positive controls—equine dermal (ED) cells infected with Jan-E EHV-1 (Fig. 2). During Jan-E EHV-1 infection, a cytopathic effect (CPE) was visible as early as 24 h p.i. and manifested by drastically changed morphology and degeneration of cells, which led to focal degeneration (Fig. 2b–f). Between 24 and 38 h p.i., a significant decrease from 98.35 to 29.02% in the level of confluence was noticed (Fig. 2g).

**Fig. 3:**
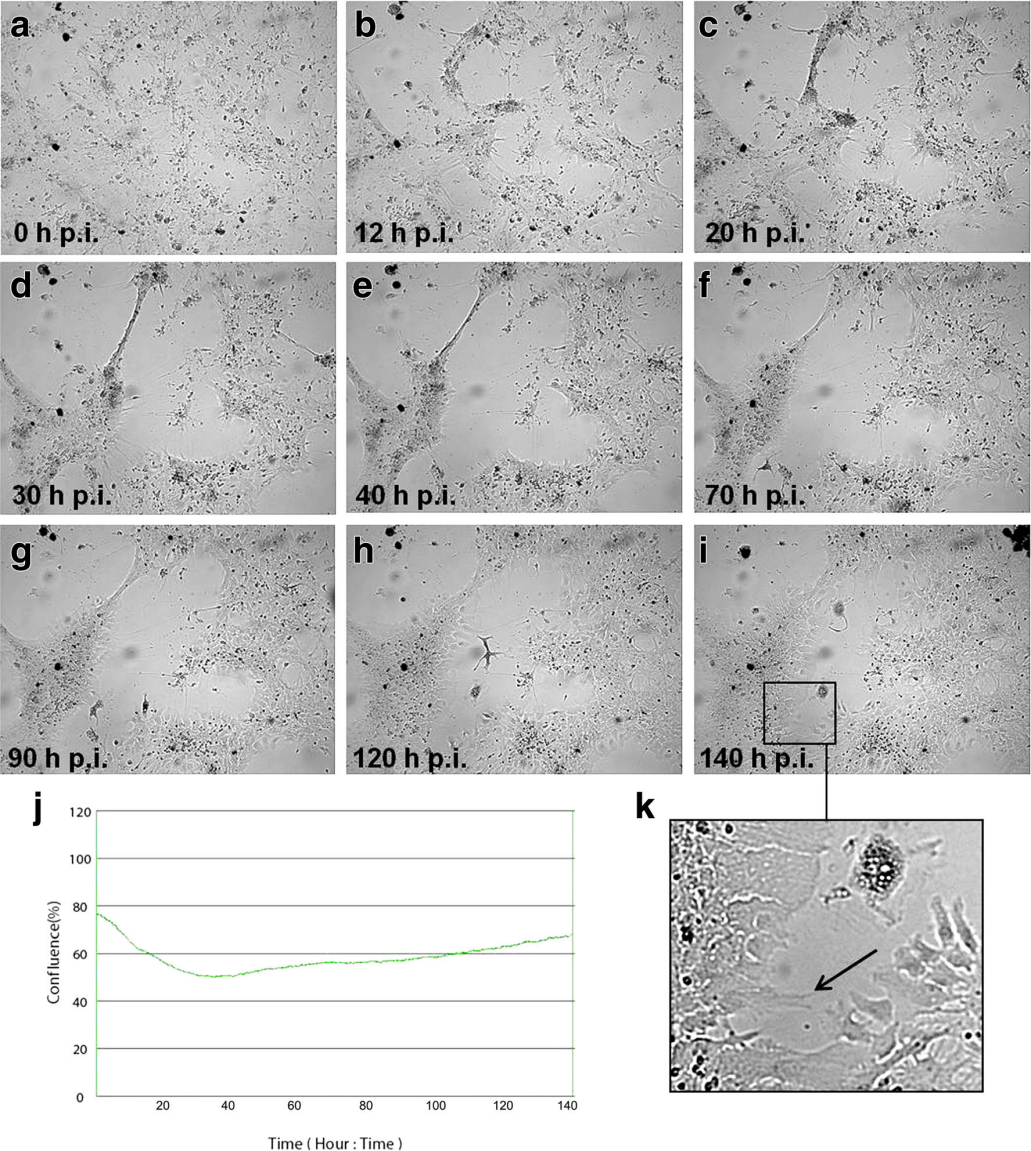
Morphological changes of primary murine neurons infected with Jan-E EHV-1 strain. Neurons were observed for 140 h through live image move analyzer JuLI™ Br (**a–i**). CPE was manifested by cell destruction and focal degeneration, confirmed by growth curve (**j**), but not so evident as in ED cells infected with Jan-E EHV-1. At the free surface inside the plaques, single cells were observed (**k**, *arrow*). Objective magnification ×40

In neurons infected with Jan-E strain, destruction of cells and focal degeneration was also observed, however not so evident as in ED cells (Fig. 3). At the beginning, confluence of the culture decreased from 79% (0 h p.i.) to 51.73% (33 h p.i.) and then gradually began to increase up to 68.64% in 140 h p.i. (Fig. 3j). Changes in the morphology of infected neurons were apparent, but some of them remained unchanged and retained their projections (Fig. 3c–h). Moreover, at the empty surface inside the plaques, new single cells were identified (Fig. 3i, k; arrow).

**Fig. 4:**
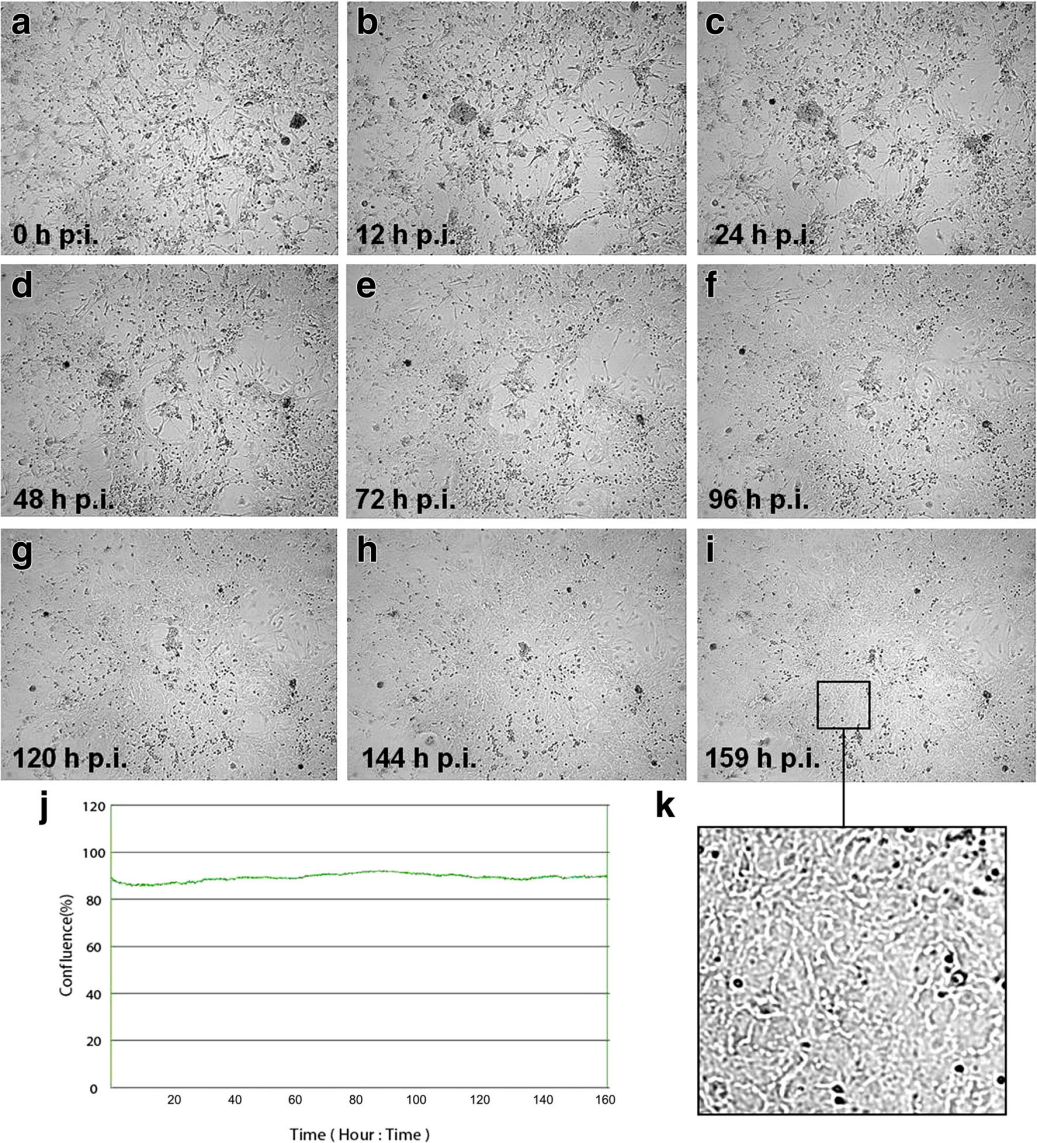
Morphological changes of primary murine neurons infected with Rac-H EHV-1 strain. Neurons were observed for 160 h through live image move analyzer JuLI™ Br (**a–i**). CPE was not as obvious as in neurons infected with Jan-E EHV-1 strain. There was no change in the level of confluence (**j**); however, rounding of the infected cell and fusion with adjacent cells to form syncytia was visible (**k**). Objective magnification ×40

In neurons infected with Rac-H strain, CPE was not as clearly visible as in neurons infected with Jan-E EHV-1 strain. Even though there was no change in the confluence of the culture (Fig. 4j), the differences in cell morphology were visible. CPE was manifested by rounding of the infected cell and fusion with adjacent cells to form syncytia (Fig. 4a–i). However, infected cells did not undergo lysis even at 160 h p.i.

**Fig. 5:**
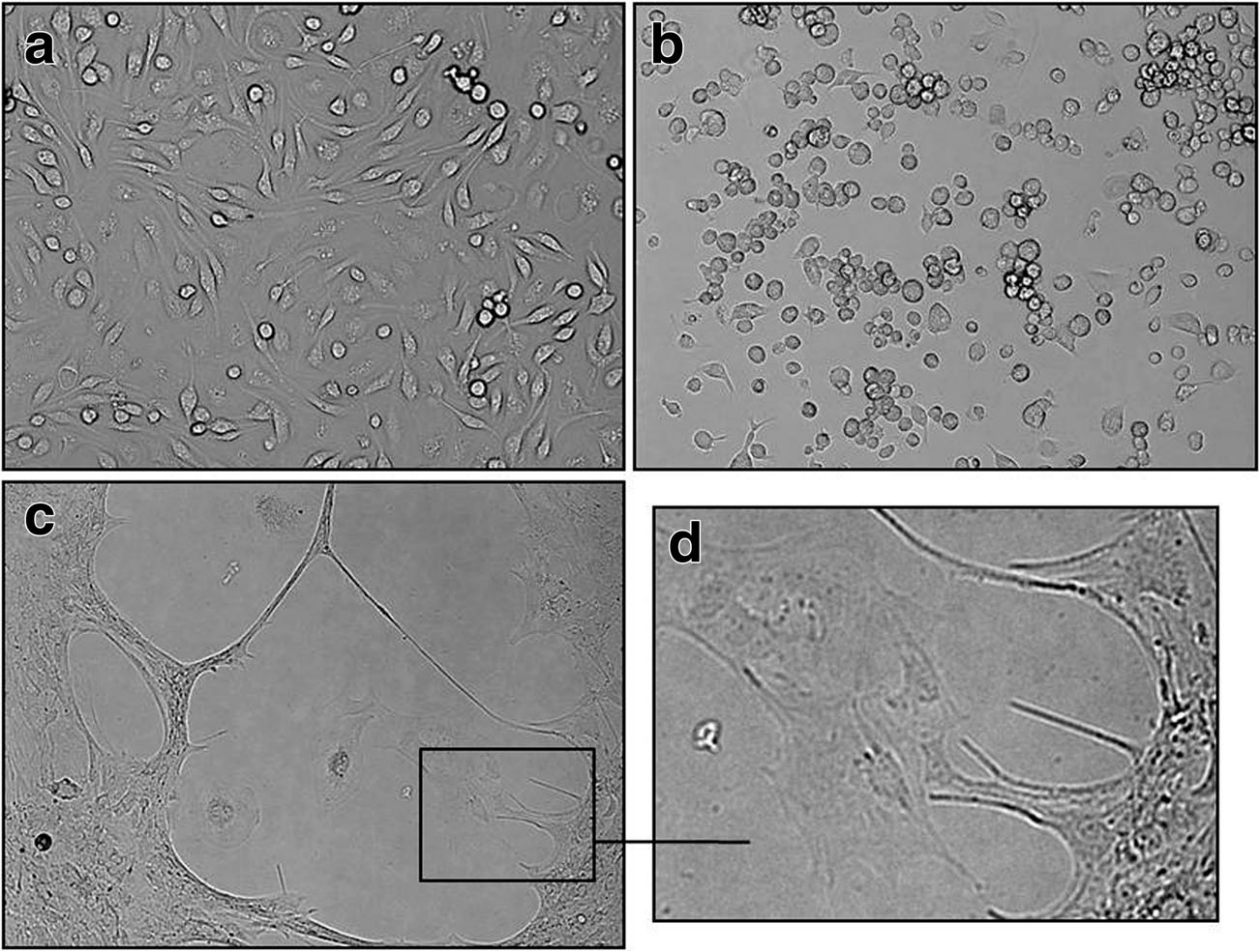
CPE in primary murine neurons infected with EHV-1 strains (8 weeks p.i.). Microscope image of uninfected neurons (**a**) and cells infected with Jan-E (**b**) and Rac-H (**c**) EHV-1. After that time, single neurons within the plaque were still observed (**d**). Magnification ×200

In order to determine how long neurons were able to survive, they were left infected with Jan-E or Rac-H EHV-1 for 8 weeks. After that time, neurons were still viable and CPE was not significantly different from this observed in 160 h p.i. In cultures infected with Jan-E strain, cells were rounded and devoid of neuronal projections (Fig. 5b). On the other hand, in neuronal culture infected with Rac-H strain, focal degeneration (Fig. 5c) as well as neurons within the plaque were observed (Fig. 5d).

### EHV-1 replication during long-term infection of primary murine neurons

**Fig. 6:**
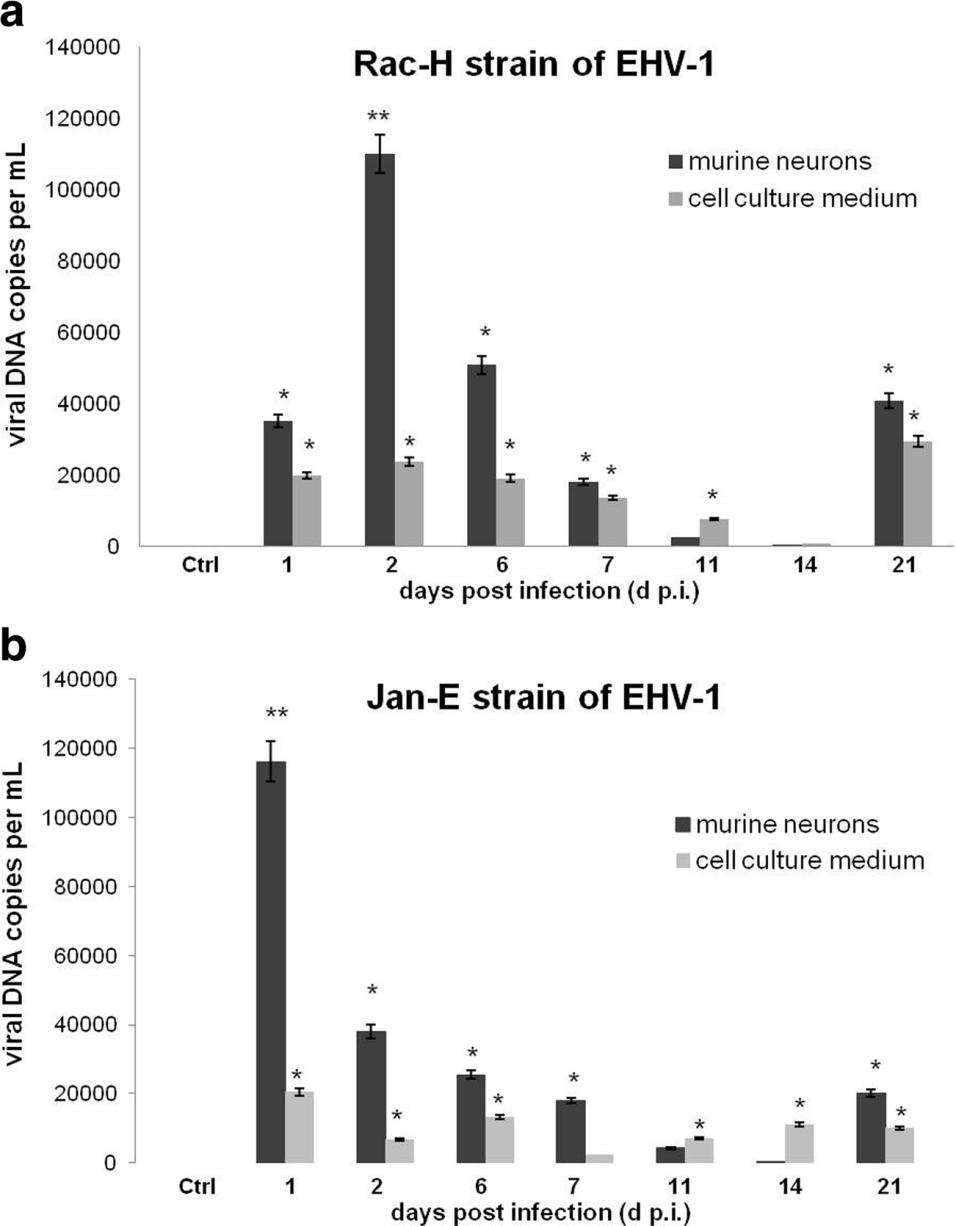
Real-time PCR analysis of viral DNA level during long-term neurons infection. Comparison of viral DNA level (viral gB copy number per mL) in primary murine neurons and cell culture medium during 21 days post infection with Rac-H (**a**) and Jan-E (**b**) strains of EHV-1. Statistical differences were interpreted as significant at *P* ≤ 0.05 (*), highly significant at *P* ≤ 0.01 (**) and not significant at *P* ≥ 0.05

Real-time PCR was applied to detect viral DNA in neuronal cells and in neuroculture medium during long-term infection with Jan-E and Rac-H EHV-1 strains (1, 2, 6, 7, 11, 14 and 21 days p.i.). During infection of neurons with Rac-H reference strain, a significant decrease of copy number of viral DNA was observed from 24 h p.i. (2.94 ± 0.59 × 10^4^) to 7 days p.i. (1.28 ± 0.89 × 10^4^) (Fig. 6a). In comparison to the uninfected control, the highest copy number of viral DNA was observed at 48 h p.i. (1.0 ± 0.27 × 10^5^; *P* ≤ 0.01). Subsequently, 11 and 14 days p.i., a decrease in the copy number of viral DNA was observed in comparison to the first 24 h of infection (from 1.70 ± 0.68 × 10^3^ to 1.69 ± 0.32 × 10^2^). Significantly, after 21 days p.i., the copy number of viral DNA increased to the level observed at 24 h p.i. (3.15 ± 1.57 × 10^4^). In order to determine the dynamics of replication of Rac-H strain in primary murine neurons, the copy number of viral DNA released to cell culture medium was examined starting from 24 h p.i. The results showed that the levels of viral DNA released to cell culture medium during long-term infection of neurons with Rac-H strain remained comparatively constant starting from 24 h p.i. (from 1.41 ± 1.09 × 10^4^ in 24 h p.i. to 2.24 ± 0.92 × 10^4^ in 21 days p.i.; *P* ≤ 0.05). However, at 11 and 14 days p.i., a decrease of the viral DNA level in the culture medium was observed (from 5.82 ± 1.08 × 10^3^ to 5.42 ± 0.81 × 10^2^).

After infection with Jan-E strain of EHV-1, the highest copy number of viral DNA was observed 24 h p.i. During the course of infection, we observed a decrease in viral DNA copy number reaching the lowest levels at 11 and 14 days p.i. (from 3.31 ± 1.27 × 10^3^ to 2.86 ± 0.51 × 10^2^). Afterwards, at 21 days p.i., the copy number of viral DNA increased (1.53 ± 0.89 × 10^4^) and was detected at a level comparable to the level detected at 24 h p.i. The copy number of viral DNA in culture medium remained at a constant level throughout the course of infection, ranging from 24 h p.i. to 21 days p.i. (from 1.56 ± 0.93 × 10^4^ to 1.1 ± 1.39 × 10^4^; *P* ≤ 0.05).

### The effects of EHV-1 passage in primary murine neurons

**Fig. 7:**
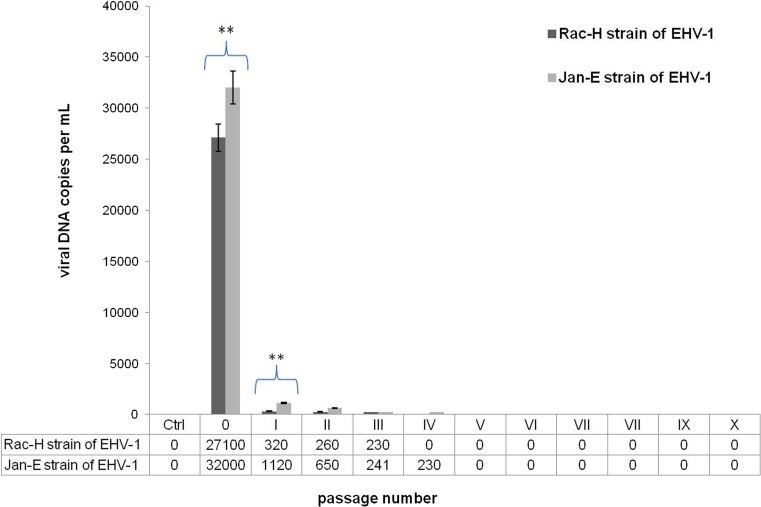
Real-time PCR analysis of viral DNA level during multiple passages of EHV-1 strains. The neurons-to-neurons EHV-1 (Rac-H and Jan-E strain) passage was repeated ten times. Significant level of viral DNA (viral gB copy number per mL) was observed only in the zero (0) and the first (I) passages. Statistical differences were interpreted as significant at *P* ≤ 0.05 (*), highly significant at *P* ≤ 0.01 (**) and not significant at *P* ≥ 0.05

**Fig. 8:**
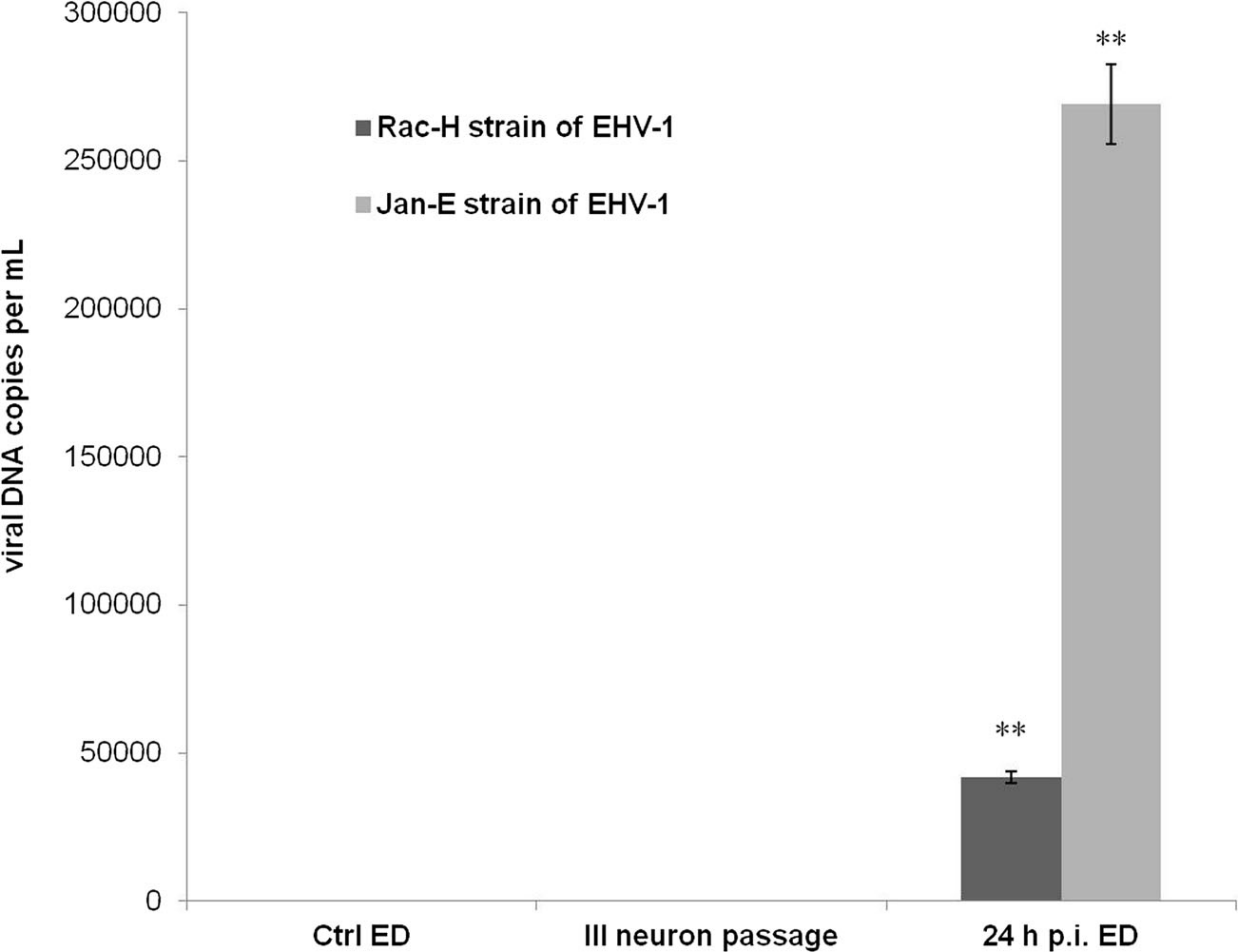
ED cells co-cultivated with neuroculture medium from the third passage. Real-time PCR analysis of viral DNA level (viral gB copy number per mL) in ED cells infected with the neuroculture medium from the third passage (III) of EHV-1 in neurons. Statistically significant (*P* ≤ 0.01, **) increase in the copy number of viral DNA for both Rac-H and Jan-E strain was observed in ED cells after 24 h p.i.

The results of passaging Rac-H and Jan-E strains of EHV-1 in primary murine neurons showed that only in the zero passage (0), which simultaneously constituted a positive control, a statistically significant increase in the copy number of viral DNA (Rac-H 2.71 ± 1.02 × 10^4^; Jan-E 3.20 ± 1.12 × 10^4^; *P* ≤ 0.01) was observed in comparison to uninfected control (Fig. 7). In the first passage (I), EHV-1 replication was also observed; however, the copy number of viral DNA was significantly lower than in the zero passage (Rac-H 3.20 ± 0.14 × 10^2^; Jan-E 1.12 ± 0.42 × 10^3^; *P* ≤ 0.05). In the second passage (II), viral DNA was still detected in neurons; however, the results were not statistically significant in comparison to the uninfected control. Interestingly, beginning from the fourth passage (IV) (Rac-H and Jan-E) to the tenth passage (X), viral DNA was not present in neurons at a detectable level. For that reason, we decided to infect equine dermal (ED) cells with the neuroculture medium from the third passage (III) of EHV-1 in neurons (Rac-H 230 ± 0.49 copies per mL; Jan-E 241 ± 0.51 copies per mL). After 24 h of incubation, a statistically significant increase in the copy number of viral DNA for both Rac-H and Jan-E strains was observed in ED cells in comparison to neurons from the third passage (Rac-H 4.17 ± 1.02 × 10^4^; Jan-E 2.69 ± 1.51 × 10^5^; *P* ≤ 0.01) (Fig. 8).

## Discussion

The problem with selection of proper experimental model should be strongly emphasized in the context of the research on EHV-1 infections. As we mentioned in our previous paper, the use of natural host is very limited due to the difficulties associated with finding “immunologically naive” test animals that have not been in contact with the virus (Cymerys et al. 2010). Pusterla and Hussey (2014) indicated that more than 80% of horses are estimated to be latently infected with EHV-1. Moreover, other equine herpesviruses, such as EHV-2 or EHV-5, may affect the results of the experiment (Borchers et al. 1999; Ruszczyk et al. 2001; Osińska et al. 2012). For that reason, BALB/c mouse strain in which EHV-1 infection is similar to that in the natural host constitutes a suitable research model (Bańbura et al. 2000; Awan et al. 1990; Gosztonyi et al. 2009; Walker et al. 1999). Results presented by the other authors provide a strong evidence that EHV-1 causes latent infection in mice with the presence of viral DNA not only in mononuclear cells but also in neurons of the olfactory bulbs and trigeminal ganglion (Bańbura et al. 2000; Baxi et al. 1995; Marshall and Field 1997). Therefore, it is crucial to clarify what kind of processes take place during the neurological episode, especially during long-term infection with EHV-1. For that reason, presented here in vitro model utilizing cultured primary murine neurons provides a simple and effective method to examine the kinetics of EHV-1 replication and to determine differences between EHV-1 strains and allows to investigate the specific virus-neuron relationship, including long-term EHV-1 infection. Using real-time cell growth analysis, during the early stages of infection, we observed the occurrence of CPE in primary murine neuron culture after infection with both Jan-E and Rac-H strains of EHV-1 (Figs. 3 and 4). These changes were best characterized by abnormal cell morphology, e.g., shrunken, rounded and distorted appearance. CPE is typical for cells which undergo lysis due to viral infection and that indicate productive infection. Jan-E (field strain) induced CPE within 48 h after infection of murine neuronal cells. On the other hand, infection with Rac-H (reference strain) led to similar result within 96 h. These findings were confirmed by real-time PCR results, which showed the largest copy number of viral DNA in primary murine neurons at 24 and 48 h p.i. for Jan-E strain and 48 and 96 h p.i. in the case of Rac-H strain (Fig. 6). We previously confirmed the presence of EHV-1 antigen within neurons, using immunofluorescence staining, and also proved that EHV-1 can be transported from neuron to neuron (Cymerys et al. 2010; Słońska et al. 2014). After infection of primary murine neurons with Jan-E strain, rearrangements of the actin distribution and formation of long, thin or short, wide actin-containing cell projections were observed. These projections stretched from cell to cell contributing to direct spread of virus particles to adjacent cells without release from the cell. In the case of Rac-H infection, the formation of long projections was not observed (Słońska et al. 2014). However, it should be noted that the CPE caused by Rac-H in primary murine neurons has a different form than that caused by Jan-E strain. Rac-H does not cause the formation of large free spaces in cell culture, the cells remain closely arranged, which also allows for the transport of the virus from cell to cell (Fig. 4). Interestingly, the differences in actin cytoskeleton rearrangement and the associated direct transport of virions between cells were also partially reflected in the results of real-time PCR. Both for Jan-E and Rac-H strains, the copy number of viral DNA was higher in cells than in the cell culture medium, but apparently for the Rac-H strain, more progeny virions were released from cells than in the case of Jan-E strain (Fig. 6).

In our previous studies, we demonstrated that, despite CPE occurrence, the infection of murine neurons with either of EHV-1 strains (Rac-H or Jan-E) induced changes associated with apoptotic cell death such as DNA fragmentation, chromatin condensation, membrane blebbing and cell shrinkage (Cymerys et al. 2012). However, despite evident changes in the morphology of Jan-E infected neurons, some of them remained unchanged and retained their neuronal projections (Fig. 3c–h). Moreover, at the empty surface inside the plaques, single neurons were identified (Fig. 3i, k; arrow). In neurons infected with Rac-H strain, CPE was manifested by rounding of the infected cell and fusion with adjacent cells to form syncytia (Fig. 4a–i), but the cells did not undergo lysis even at 166 h p.i. Some neurons in EHV-1 infected cultures changed their shape and degenerated, while some remained morphologically unchanged. The former were presumed to undergo lytic infection with the virus. The latter may have been resistant to EHV-1 infection, or EHV-1 infection was either abortive or latent in such neurons. Further investigation using in situ labelling of viral proteins (IHC or IF) or nucleic acids (ISH) would be necessary to distinguish between these possibilities. These findings confirmed our previous studies, in which we demonstrated that 60–80% of neurons did not undergo lysis despite the infection (Cymerys et al. 2012) and we suggested that EHV-1 is responsible for suppression of apoptosis in neurons. The question remains, what mechanisms allow neurons to survive during long-term EHV-1 infection. According to our previous research, which revealed the inhibition of apoptosis in EHV-1-infected neurons, we concluded that the control of apoptosis may be the key mechanism regulating the balance between productive and latent infection at the site of virus persistence; however, this hypothesis requires further investigation (Cymerys et al. 2012).

In the present study, we also compared the neurovirulence of Rac-H and Jan-E EHV-1 strains during multiple passages in neuronal cell culture. The results showed that multiple passages of EHV-1 in neurons lead to the inhibition of viral replication as early as in the third passage (Fig. 7). Interestingly, the inhibition of the EHV-1 replication occurred exclusively in neurons, because the equine dermal cells (ED cell line) infected with neuroculture medium from the third passage showed the presence of large number of viral particles capable of replication (Fig. 8). These results may suggest that even though we have not confirmed viral replication by real-time PCR, virus was apparently present in neurons. This may also suggest that the number of viral particles is insufficient to maintain infection in murine neurons but sufficient for replication in highly susceptible ED cells. It is also noteworthy that during real-time PCR analyses viral DNA (late gene—gB) was detected (from 24 h p.i. to 21 days p.i.) (Fig. 6).

In conclusion, our results suggest that certain balance between EHV-1 and neurons has been established during in vitro infection allowing neurons to survive long-term infection. It seems to be logical since neurons are the site of latency, and for the virus benefit, they should survive to maintain latent virus. Using real-time cell growth analysis, we have demonstrated for the first time that primary murine neurons are able to survive long-term EHV-1 infection; nevertheless, the question of interactions between the equine alphaherpesviruses and neuronal cells is still pending, and further studies on EHV-1 replication in neurons are necessary.
